# The fructose-1,6-bisphosphatase deficiency and the p.(Lys204ArgfsTer72) variant

**DOI:** 10.1590/1678-4685-GMB-2020-0281

**Published:** 2021-05-14

**Authors:** Franciele Cabral Pinheiro, Rodrigo Ligabue-Braun, Ana Cecília Menezes de Siqueira, Camila Matuella, Carolina Fischinger Moura de Souza, Fabíola Paoli Monteiro, Fernando Kok, Ida Vanessa Doederlein Schwartz, Fernanda Sperb-Ludwig

**Affiliations:** 1 Universidade Federal do Rio Grande do Sul (UFRGS), Programa de Pós-Graduação em Genética e Biologia Molecular, Porto Alegre, RS, Brazil.; 2 Hospital de Clínicas de Porto Alegre (HCPA), Centro de Pesquisas Experimentais, Porto Alegre, RS, Brazil.; 3 Universidade Federal do Pampa (UNIPAMPA), Itaqui, RS, Brazil.; 4 Universidade Federal de Ciências da Saúde de Porto Alegre (UFCSPA), Departamento de Farmacociências, Porto Alegre, RS, Brazil.; 5 Instituto de Medicina Integral Professor Fernando Figueira (IMIP), Centro de Erros Inatos do Metabolismo (CETREIM), Recife, PE, Brazil.; 6 Hospital de Clínicas de Porto Alegre, Serviço de Genética Médica, Porto Alegre, RS, Brazil.; 7 Mendelics Genomic Analysis, São Paulo, SP, Brazil.; 8 Universidade de São Paulo, Faculdade de Medicina, Departamento de Neurologia, São Paulo, SP, Brazil.

**Keywords:** Inborn error of fructose metabolism, NGS, FBPase deficiency, computational analysis, *in silico* analysis

## Abstract

Fructose-1,6-bisphosphatase (FBPase) deficiency is a rare inborn error of fructose metabolism caused by pathogenic variants in the *FBP1* gene. As gluconeogenesis is affected, catabolic episodes can induce ketotic hypoglycemia in patients. *FBP1* analysis is the most commonly used approach for the diagnosis of this disorder. Herein, a Brazilian patient is reported. The proband, a girl born to a consanguineous couple, presented with severe hypoglycemia crisis in the neonatal period. At the age 17 months, presented a new crisis accompanied by metabolic acidosis associated with a feverish episode. Genetic analysis was performed by next-generation sequencing (NGS), identifying the NM_000507.3:c.611_614del variant in homozygosis in the *FBP1* gene. *In silico* analysis and 3D modeling were performed, suggesting that this variant is associated with a loss of sites for substrate and Mg^2+^ binding and for posttranslational modifications of FBPase. The c.611_614del variant is located in a repetitive region of the *FBP1* gene that appears to be a hotspot for mutational events. This frameshift creates a premature termination codon in the last coding exon which escapes the nonsense-mediated decay mechanism, according to *in silico* analysis. This variant results in an intrinsically disordered protein with loss of substrate recognition and post-translational modification sites.

Fructose-1,6-bisphosphatase (FBPase, EC 3.1.3.1) deficiency (OMIM #229700) is a rare inborn error of fructose metabolism that presents recessive autosomal inheritance caused by pathogenic variants in the *FBP1* gene (ID:2203). Patients with this disorder fail to convert fructose-1,6-bisphosphate into fructose-6-phosphate and inorganic phosphorus, a key reaction of gluconeogenesis that occurs in the liver and kidneys. The first symptom presented is hypoglycemia associated with catabolic episodes such as prolonged fasting or high fever. In addition, other biochemical manifestations include elevated blood lactate, ketonuria and metabolic acidosis (Steinmann et al., 2012). The treatment consists of decreasing fructose intake and avoiding long-term fasting. Early diagnosis and treatment result in good prognosis for patients with FBPase deficiency (Pinto et al., 2018).

Currently, the diagnosis is usually provided by genetic analysis of the *FBP1* gene, which is located on chromosome 9q22.32. The gene spans approximately 31 kb and includes seven exons, that are translated into an enzyme comprising 338 amino acids and are expressed in the liver, intestine and kidneys (El-Maghrabi et al., 1995). The enzyme is modulated by cofactors (mono and divalent cations) and inhibitors (AMP and fructose-2,6-phosphate) that stabilize the conformation active or inactive of the protein, respectively (Shi et al., 2013). The Human Gene Mutation Database (HGMD^®^, http://www.hgmd.cf.ac.uk/ac/gene.php?gene=FBP1) (Stenson et al., 2017) describes 59 different variants involved in the pathogenesis of this disorder, including the NM_000507.4:c.472C>T, NP_000498.2:p.(Arg158Trp); NM_000507.3:c.958G>A, NP_000498.2:p.(Gly320Arg) and NM_000507.3:c.986T>C, NP_000498.2:p.(Leu329Pro) variants detected in Brazilian patients (Pinheiro et al., 2019). The variant p.(Arg158Trp) presents a frequency of 0.00003668 in gnomAD database (Karczewski et al., 2020). However, other variants detected in Brazilian patients are absent of gnomAD (Karczewski *et al.,* 2020) and AbraOm (Naslavsky et al., 2017) databases. 

This study was approved by the Research Ethics Committee of Hospital de Clínicas de Porto Alegre (project no. 17-0450), and the mother provided written informed consent for participation and publication.

The proband is a girl born to consanguineous healthy Brazilian parents at the 40^th^ week after an uneventful pregnancy and natural delivery. The birth weight was 2.8 kg. In the first 24 hours, she presented symptomatic hypoglycemia and was admitted to an intensive care unit (ICU) for three days. The child’s development was normal until 1 year and 5 months of life, when she presented a new hypoglycemic crisis associated with a febrile episode and dyspnea. She was admitted to a hospital presenting metabolic acidosis and seizures; bronchopneumonia was diagnosed, and the patient remained hospitalized for 10 days.

Three months later, the child was referred to a Reference Center for Inborn Errors of Metabolism (IEM), and a next-generation sequencing (NGS) panel for 116 treatable IEM was requested (Agilent Mendelics Custom Panel V3). This panel includes genes for the most frequent causes of monogenic recurrent hypoglycemia/metabolic acidosis, such as organic acidurias, hepatic glycogenosis, beta oxidation defects and IEM of fructose metabolism. NGS revealed that the patient was homozygous for the NM_000507.3:c.611_614del, NP_000498.2:p.(Lys204ArgfsTer72), variant in the *FBP1* gene, and a specific diet for FBPase deficiency was started. Despite the difficulties in maintaining the diet, the patient remains well, with normal cognitive development and no other episodes of decompensation (Table 1).

**Table 1 -:** Biochemical findings in the follow-up of the reported patient.

	1 y 9 m	2 y 3 m	2 y 8 m	Reference Value
Weight (kg)	15.2	15.7	12.8	-
Stature (cm)	82	88	94	-
ALT (U/L)	20	16	31	0-35
AST (U/L)	46	35	43	0-35
Glucose (micromol/L)	78	65	80	70-105
Lactate (mmol/L)	-	1.9	-	0.63-2.44
pH	-	7.38	7.34	7.38-7.44
Total Cholesterol	182	188	165	150-199
*Low Density Lipoprotein* (mmol/L)	114	118	113	≤ 130
*High Density Lipoprotein* (mmol/L)	52	48	51	≥ 40
Serum triglycerides (mg/dL)	178	292	132	< 250
Uric acid (mg/dL)	≤6.0	3.9	4.0	2,5-8

To validate the NGS findings, genomic DNA samples were obtained from the saliva of the patient and her mother using prepIT^®^∙L2P (Origene) according to the manufacturer’s guidelines. The father declined testing. Sanger sequencing of the *FBP1 gene* was performed on the ABI 3500 Genetic Analyzer (Applied Biosystems). Sequence analyses were performed in Chromas 2.6.1 software (Technelysium) and in BLAST (NCBI). The reference sequence was NM_000507.3. American College of Medical Genetics and Genomics (ACMG) recommendations were used to predict the pathogenicity of the variant (Richards et al., 2015).

The characterization of the variant included database frequency (gnomAD v2.1.1 and AbraOM), pathogenicity predictors (SIFT Indel and Mutation Taster), and a nonsense mRNA decay predictor NMDEscPredictor (Coban-akdemir et al., 2018). The Translate tool ExPASy (https://web.expasy.org/translate/) was used to translate the sequence of the mutant cDNA into the predicted amino acid sequence. The structural modeling of the wild-type (WT) and mutated FBPase structures was performed using the I-TASSER package (Zhang, 2008; Yang et al., 2014). Visualization of the structures was performed in PyMOL software (The PyMOL Molecular Graphics System, Version 1.8 Schrödinger, LLC). Functional sites were identified based on UniProt entry P09467 (The UniProt Consortium, 2019). Molecular properties were calculated with ProtParam (Gasteiger et al., 2005) for molecular weight, theoretical pI, and number of charged residues; ProteinVolume 1.3 (Chen et al., 2017) for molecular volume estimation; and GETAREA (Fraczkiewicz and Braun, 1998) for molecular area estimation. Protein motif detection was performed with ScanProsite (de Castro *et al.,* 2006); protein disorder propensity was assessed with PrDOS (Ishida and Kinoshita, 2007); and structural description was carried out with PROCHECK (Laskowski, 1993).

Sanger sequencing confirmed the genotype of the patient and showed the mother is a carrier of the variant c.611_614del, p.(Lys204ArgfsTer72) (Figure 1A). According to the ACMG criteria PVS1_strong, PP1_strong, PM2 and PM3, the variant was classified as pathogenic.

This variant is present in gnomAD (v2.1.1) in two alleles from South Asian controls with a frequency of 7.954e-6 and predicted to result in a transcript with a premature termination codon (PTC) located 72 codons after the first bp deletion (Figure 1B, C). The SIFT Indel and Mutation Taster software programs predict this allele as damaging (score 0.858) and disease causing, respectively. NMDEscPredictor showed that the mutant transcript escapes nonsense-mediated decay (NMD).

**Figure 1 -:**
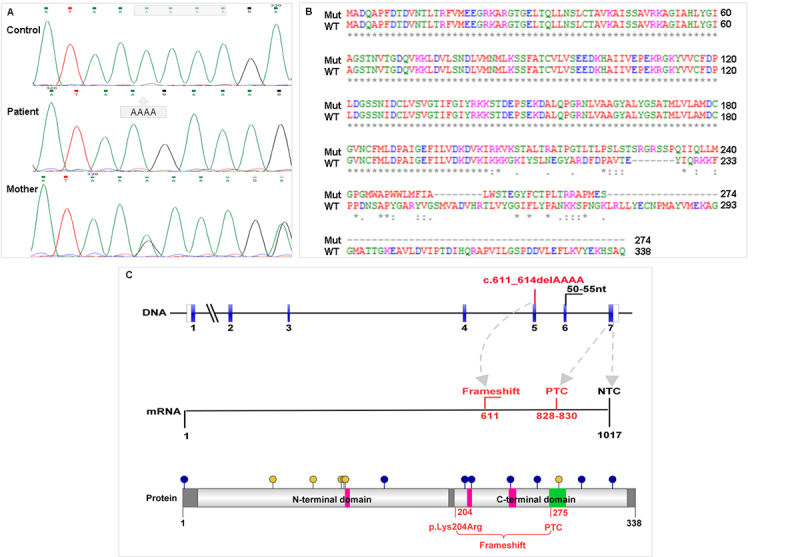
Sequences analysis of the *FBP1* gene and fructose-1,6-bisphosphatase. A) Electropherogram of control, patient and her mother evidencing the affected region in the *FBP1* gene. The control sample is a normal homozygous to the analyzed region; in their electropherogram, the gray rectangle indicates the deleted nucleotides in the patient. Highlighted in the second electropherogram, the position of the nucleotides deleted in a homozygous patient to the variant NM_000507.3:c.611_614del in *FBP1* gene. The mother's electropherogram evidences that she is a carrier of the variant in analysis. B) Amino acid sequence of the FBPase resulting from the pathogenic variant (mut) in comparison with the wild type (WT). C) Diagram representing *FBP1* DNA, *FBP1* mRNA, and FBPase variant. In DNA, the blue boxes indicate the coding exons and the region of 50-55 nucleotides upstream of the last intron that is susceptible to scaping NMD is indicated. Red indicates the variant position in DNA and its consequences in mRNA and FBPase protein. Gray arrows refer to the start of the frameshift, PTC (premature terminator codon) and NTC (normal terminator codon) between DNA and mRNA. In the protein diagram, blue dots indicate modified amino acids; yellow dots indicate metal binding sites; in pink the substrate binding regions and in green the active site. RefSeq: NM_000507.3 and P09467. The FBPase domains were named according Pfam.

The *in silico* analysis showed that the variant resulted in a protein with altered molecular properties, including an increased surface area (Table 2 and Figure 2B) and a greater propensity for the disorder than wild-type FBPase (Figure 2A, B). The results indicated the possible loss of post-translational modification sites: two N-myristoylation sites (293-298, 294-299), two serine phosphorylation sites (211, 321), and one threonine phosphorylation site (298) (Figure 1C). However, a new threonine phosphorylation site has been proposed (226).

**Table 2 -:** Comparison of physical chemical properties of wild-type and NP_000498.2:p.(Lys204ArgfsTer32) mutant fructose-1,6-bisphosphatase.

Properties	Wild-Type	Mutant
Number of amino acids	338	274
Molecular weight (kDa)	36.8	29.7
Theoretical pI	6.54	7.53
Negatively charged residues	41	28
Positively charged residues	40	29
Volume (Å³)	43,909	34,705
Area (Å²)	16,164	16,352

**Figure 2 -:**
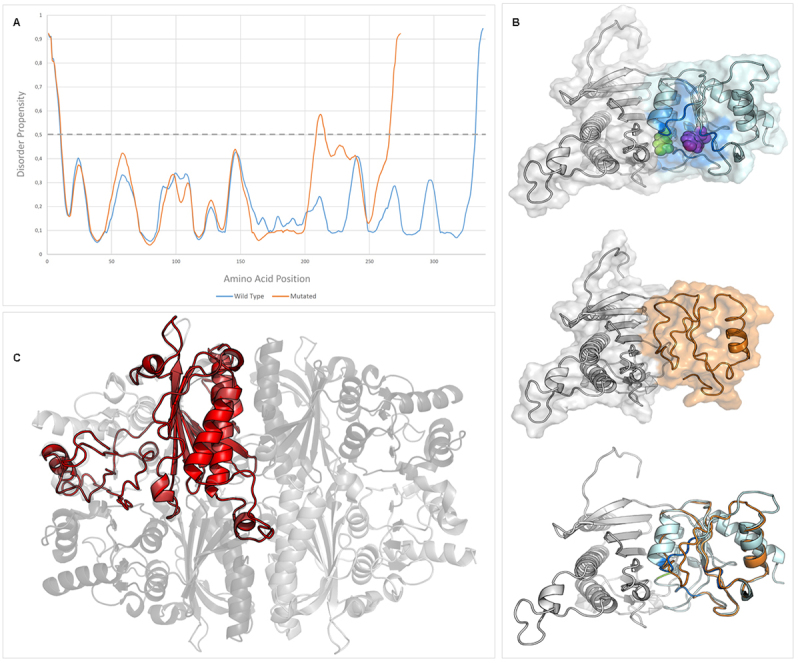
*In silico* analysis of the NP_000498.2:p.(Lys204ArgfsTer32) variant in the *FBP1* gene. A) Disorder prediction for wild-type (blue line) and mutated (orange line) FBPases. B) 3D modeling of fructose-1,6-bisphosphatase. First image, the wild-type FBPase. The region affected in the mutant protein is shown in light blue, the altered substrate binding regions are shown in dark blue, the altered substrate recognition site is shown in magenta, and the altered Mg binding site is shown in green. Right below, the mutant FBPase. The region affected by the frameshift mutation is shown in orange. All sites highlighted in WT are absent in the mutant protein. Lastly, the superposition of WT and mutant FBPase, colored according to the previous. C) The structure of fructose-1,6-bisphosphatase tetramer. Superposition of mutant FBPase monomer (red) onto the functional FBPase tetramer (in different shades of gray). Note the absence of structured elements in the exposed region of the monomer. Tetramer information from PDB ID 2FIE (Lai et al., 2006).

We report herein a case of a Brazilian patient with a pathogenic variant (c.611_614del) previously found in homozygosity in one Pakistani (Ijaz et al., 2017) and in two compound heterozygous Indian patients (Bhai et al., 2018; Sharma et al., 2018) with FBPase deficiency. This is the first time this genotype has been described in a Brazilian patient. In a previous study with six patients from southern Brazil, three different variants were detected (Pinheiro et al., 2019), showing the genetic heterogeneity of FBPase deficiency in the country. Until this moment, the Brazilian patients are the only reports of FBPase deficiency in South America.

The c.611_614del allele is located in a repetitive region of exon 5 (NM_000507.3) of the *FBP1* gene (5’-GAAGATAAAAAAGAAATAAAAT-3’). This sequence favors DNA polymerase slipping during replication and the appearance of deletions/insertions in the nucleotide sequence. Therefore, this could be considered a hot spot region; most likely, this allele emerged independently in the three families previously reported (Ijaz et al., 2017; Bhai et al., 2018; Sharma et al., 2018). In addition, c.611_614del is present in the gnomAD database only in South Asian controls. The distribution of this allele reinforces the hypothesis that this may be a pan-ethnic allele, such as the most common pathogenic variant in *FBP1,* the NM_000507.3:c.959dup allele (Kikawa et al., 1997).

The deletion of four nucleotides in *FBP1* gene results in a premature termination codon (PTC) in the last coding exon (codon 275) (Figure 1C). The presence of a PTC can activate the NMD mechanism, so we analyzed this possibility with the NMDEscPredictor software. The results indicate that c.611_614del allele escapes from NMD. In this sense, it is important to point out that NMD exhibits variability in its efficiency across transcripts, cells, tissues, and individuals in both physiological and pathological contexts, and nonsense variants can escape NMD via many routes. When the PTC is close to the natural terminator codon (NTC), such as in this case report (Figure 1C), the presence of polyA binding protein (PABPC1) inhibits the UPF1 binding, the core of NMD substrate recognition, resulting in efficient translation termination (Dyle et al., 2020). If the mRNA escapes from the NMD pathway, the protein generated is shortened and frameshifted, probably without any enzymatic activity, as was observed in frameshift variants in *POMP* gene causing immune dysregulatory syndrome (Poli et al., 2018). However, no functional assay was performed to verify if the mutant transcript escapes NMD. Besides that, FBPase activity was not measured in our patient or in the Pakistani and Indian patients (Ijaz et al., 2017; Bhai et al., 2018; Sharma et al., 2018). The FBPase is mainly expressed in the liver, kidney and intestine (Uhlén et al., 2015), so the analysis of enzyme activity for the reliable diagnosis of FBPase deficiency requires a liver biopsy, an invasive procedure that should be avoided. 

FBPase is a 37 kDa homotetramer whose activity is modulated by inhibitors and inducers. The concentration of the ligands in the active and allosteric sites determines the conformational structure of the enzyme. Thus, the presence of AMP and fructose-2,6-bisphosphate induces an inactive state of FBPase. On the other hand, the fructose-1,6-bisphosphate substrate favors the active state of the enzyme. In addition, the ideal catalytic activity of FBPase depends on cofactors such as Mg^2+^ (Ke et al., 1990; Shi et al., 2013).The computational analysis showed that the mutant lacks substrate binding sites (sites 213-216 (NEGY), 244-249 (RYVGSM) and 275-277 (KLR)), in addition to other important regions as a site of linkage with Mg^2+^ (site 331, E) (Figure 2B), according to what was previously predicted by Ijaz *et al*. (2017). The refinement of the analysis showed that the mutant enzyme had a larger surface area because of the increase in coiled regions (Figure S1). This characteristic is implicated in the interactions of the mutant enzyme because the variant region is exposed to the cellular medium. Thus, the interaction of the mutant with other monomers to form the functional tetramer (Geldern *et al.,* 2006) would be less affected than interactions with other molecules (Figure 2C). In addition, the increase in coiled regions (Figure S1) induces the formation of a structurally disordered protein, influencing its stability. Disordered regions are able to bind more partners and bestow new functions to otherwise ordered proteins (Uversky et al., 2008). This is especially relevant in neurodegenerative disorders and various types of cancer (Martinelli et al., 2019).

FBPase deficiency is a severe disorder that can be fatal, mainly in newborns. Early diagnosis helps to increase the survival rate and patient quality of life. The definitive diagnosis is provided by identification of the two pathogenic alleles in the *FBP1* gene. However, the classification of variants is a challenge in clinical routine, and the *in silico* tools assist in this process. Thus, the computational approach has been used to predict the pathogenicity and molecular mechanisms involved in variants causing different human diseases (Teckan, 2016). In this sense, based on *in silico* analysis, we described the molecular alterations implicated in the pathogenesis of the c.611_614del variant, a pan-ethnic allele involved in FBPase deficiency.

## Supplementary material

The following online material is available for this article:

Figure S1 - Secondary structure comparison between wild type and mutated fructose-1,6-bisphosphatase.
