# Supplementary material for: The fructose-1,6-bisphosphatase deficiency and the p.(Lys204ArgfsTer72) variant
Source: Genet Mol Biol. 2021 May 14;44(2):e20200281. doi: 10.1590/1678-4685-GMB-2020-0281 (PMC8127874; doi:10.1590/1678-4685-GMB-2020-0281)

Genetics and Molecular Biology

https://doi.org/10.1590/1678-4685-GMB-2020-0281

Supplementary Material to "The fructose-1,6-bisphosphatase deficiency and the p.(Lys204ArgfsTer72) variant"

Figure S1: Secondary structure comparison between wild type and mutated fructose-1,6-bisphosphatase. FBPases showing the shorter length and increase in coil content for the mutated enzyme. WT=wild type; Mut=mutated.

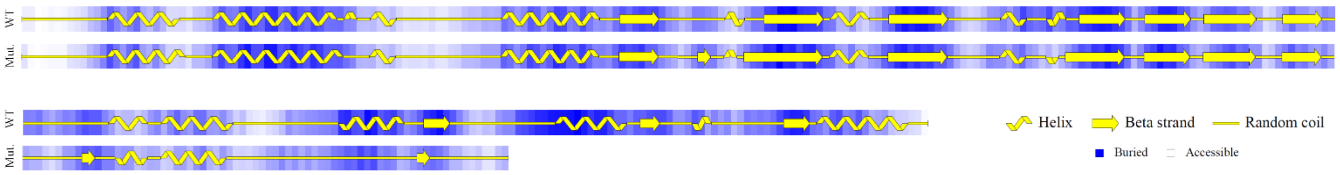

Supplement: Figure S1 - [file 1415-4757-GMB-44-2-e20200281-s1.pdf]
